# Does tocilizumab have a magical therapeutic effect on COVID-19 patients without obvious adverse reactions?

**DOI:** 10.1073/pnas.2009961117

**Published:** 2020-11-17

**Authors:** Yong Wang, Qing Mao, Xiangdong Zhou

**Affiliations:** ^a^Department of Rheumatology, Southwest Hospital, Army Medical University (the Third Military Medical University), Chongqing 400038, People's Republic of China;; ^b^Department of Infection, Taikang Tongji COVID-19 Hospital, Wuhan 430000, People's Republic of China;; ^c^Department of Infection, Southwest Hospital, Army Medical University (the Third Military Medical University), Chongqing 400038, People's Republic of China;; ^d^Department of Infection, Huoshenshan COVID-19 Hospital, Wuhan 430000, People's Republic of China;; ^e^Department of Respiratory and Critical Care Medicine, Southwest Hospital, Army Medical University (the Third Military Medical University), Chongqing 400038, People's Republic of China

Coronavirus disease 2019 (COVID-19) may cause a cytokine storm, which may lead to respiratory failure or even death, and the mortality rate for critically ill cases reached 60.5% (1, 2). In a retrospective observational study conducted by Xu et al. (3), all 21 patients, including 17 seriously ill and 4 critically ill cases (4), had been discharged after an average of 15.1 d, and no patient died after taking tocilizumab (TCZ). This indicates that TCZ has almost a magical therapeutic effect on COVID-19 patients. However, our findings are not optimistic. In Taikang Tongji Hospital, all 12 patients with elevated interleukin-6 (IL-6) levels, including 8 seriously ill and 4 critically ill, received TCZ therapy. During a 1-wk period of observation, clinical improvement was observed in nine patients, two patients finally died accompanied by a persistent drop in the corresponding lymphocyte counts, and one patient’s condition became exacerbated after TCZ treatment. In the two critically ill patients who passed away, a persistent and dramatic increase of IL-6 level was observed, which ranged from 12.84, 100.4 pg/mL to 2,932, 1,308 pg/mL, respectively, indicating that the poor prognosis of patients with COVID-19 was positively correlated with persistent elevation of IL-6 level. In addition, in Huoshenshan Hospital, five of eight critically ill patients died accompanied by a rapidly increased level of IL-6 within 1 wk after TCZ treatment. Similarly, at the Zhongfaxincheng campus of Tongji Hospital in Wuhan, three of seven critically ill patients who received only a single dose of TCZ died, while one patient had a clinical outcome of disease aggravation (5). TCZ inhibits IL-6 binding to its receptor meanwhile inhibiting IL-6 uptake and degradation within cells (6), so the increased IL-6 may be the inevitable consequence of disorder in patients who already have very high IL-6 levels. Therefore, TCZ may be effective for treating seriously ill COVID-19 patients, while it is not an ideal choice for critically ill patients.

As a kind of biological disease-modifying antirheumatic drug, TCZ may weaken the innate immunity in the body, which associates with high risk of serious infections (7, 8). In a randomized double-blind trial of TCZ in adults with systemic sclerosis, serious infections were found more common in TCZ group (7 [16%] of 43 patients) than in the placebo group (2 [5%] of 44) (8). There were no obvious adverse reactions after TCZ therapy in the published study (3). However, in our study, a 69-y-old male patient was noted with severe infection and worsened symptoms after being treated with TCZ. We speculate that TCZ treatment, especially for elderly patients with COVID-19, may increase the risk of viral and bacterial infection due to suppression of immune function. Therefore, we should be aware of the side effects of TCZ, and it is also highly essential to evaluate the efficacy and safety of TCZ by rigorous randomized controlled trials.
